# Immunoreactive peptide maps of SARS-CoV-2

**DOI:** 10.1038/s42003-021-01743-9

**Published:** 2021-02-12

**Authors:** Nischay Mishra, Xi Huang, Shreyas Joshi, Cheng Guo, James Ng, Riddhi Thakkar, Yongjian Wu, Xin Dong, Qianlin Li, Richard S. Pinapati, Eric Sullivan, Adrian Caciula, Rafal Tokarz, Thomas Briese, Jiahai Lu, W. Ian Lipkin

**Affiliations:** 1 Center for Infection and Immunity, Mailman School of Public Health, Columbia University, New York, NY USA; 2 Sun Yat-sen University, Guangzhou, Guangdong Province China; 3 Center for Infection and Immunity, Fifth Affiliated Hospital, Sun Yat-sen University, Zhuhai, Guangdong Province China; 4 School of Public Health, Sun Yat-sen University, Guangzhou, Guangdong Province China; 5 Nimble Therapeutics Inc, Madison, WI USA

**Keywords:** Infectious-disease diagnostics, SARS-CoV-2, Infection, Viral infection

## Abstract

Serodiagnosis of SARS-CoV-2 infection is impeded by immunological cross-reactivity among the human coronaviruses (HCoVs): SARS-CoV-2, SARS-CoV-1, MERS-CoV, OC43, 229E, HKU1, and NL63. Here we report the identification of humoral immune responses to SARS-CoV-2 peptides that may enable discrimination between exposure to SARS-CoV-2 and other HCoVs. We used a high-density peptide microarray and plasma samples collected at two time points from 50 subjects with SARS-CoV-2 infection confirmed by qPCR, samples collected in 2004–2005 from 11 subjects with IgG antibodies to SARS-CoV-1, 11 subjects with IgG antibodies to other seasonal human coronaviruses (HCoV), and 10 healthy human subjects. Through statistical modeling with linear regression and multidimensional scaling we identified specific peptides that were reassembled to identify 29 linear SARS-CoV-2 epitopes that were immunoreactive with plasma from individuals who had asymptomatic, mild or severe SARS-CoV-2 infections. Larger studies will be required to determine whether these peptides may be useful in serodiagnostics.

## Abstract (web summary)

Mishra, Huang et al. identify 29 linear SARS-CoV-2 epitopes that are immunoreactive with the plasma from individuals who had asymptomatic, mild, or severe SARS-CoV-2 infections. This study suggests the possibility of using these peptides to discriminate the exposure to SARS-CoV-2 and other human coronaviruses.

## Introduction

Differential serodiagnosis of human coronavirus (HCoV) exposure may be challenging due to cross-reactive immunity to SARS-CoV-2, SARS-CoV-1, MERS-CoV, OC43, 229E, HKU1, NL63^1–3^. Specific plaque reduction neutralization tests are labor-intensive, require work with live virus in high-level containment facilities, and target only neutralizing antibodies. Here we report the use of proteome-wide high-density peptide microarray (HCoV peptide array) to detect specific humoral immune responses to SARS-CoV-2 and other HCoVs.

The HCoV peptide array is a programmable microarray for epitope discovery that can accommodate up to three million distinct linear peptides on a 75-mm by 26-mm glass slide (Nimble therapeutics, Madison, USA). Each HCoV peptide array is divided into 12 subarrays, with each subarray comprising ~172,000 12-amino acid (aa) nonredundant linear peptides that tile the proteomes of known HCoVs with 11 amino acid overlap^4–7^(Supplementary Table 1). The 12-mer format is based on the observation that serum antibodies bind linear peptide sequences ranging from 5 to 9 amino acids (aa) and bind most efficiently when targets are flanked by additional amino acids^8^. A total of 132 plasma samples were tested using eleven 12-plex HCoV peptide arrays to identify 29 linear SARS-CoV-2 epitopes that were immunoreactive with plasma from individuals who had asymptomatic, mild, or severe SARS-CoV-2 infections.

## Results and discussion

We examined the immunoreactivity of 100 plasma samples collected at two timepoints from 50 COVID-19 patients with active or recent SARS-CoV-2 infection confirmed by SARS-CoV-2 qPCR of nasal swabs (Groups 1, 2, and 3). Controls included 11 patients with a history of SARS-CoV-1 infection (Group 4), 10 healthy subjects (Group 5), and 11 patients with a known history of exposure to other HCoVs (Group 6) (Supplementary Tables 2 and 3). Amongst the SARS-CoV-2 subjects were 22 COVID-19 patients with severe illness (Group 1), 22 COVID-19 patients with mild illness (Group 2), and six subjects with asymptomatic SARS-COV-2 infection (Group 3). Plasma samples were collected from COVID-19 patients at two different time points, a minimum of ~2 weeks apart, (Groups 1, 2 and 3). The first time point (early) sample was collected 12.9 ± 5.9 post onset of disease (POD) for the mild disease group, and at 9.6 ± 3.5 days POD for the severe disease group. The second time point (late) samples were collected at 34.7 ± 8.3 days POD for the mild disease group, and at 24.8 ± 6.8 days POD for the severe disease group. For the asymptomatic group, the first time point sample was collected on the day of hospitalization; the second time point sample was collected at 14.5 ± 4.6 days after the day of hospitalization. Clinical status was classified as asymptomatic, mild, and severe according to the “Diagnosis and Treatment Protocol for Novel Coronavirus Pneumonia” issued by National Health Commission of the People’s Republic of China (trial version 7)^9,10^.

Plasma samples were heat-inactivated at 56 °C for 30 min, diluted (1:50), added to HCoV peptide arrays, incubated with Alexa Fluor 647 labeled goat anti-human IgG, Cy™3 labeled goat anti-human IgM antibodies, and scanned on a microarray scanner. Fluorescence signal data for all the peptides from IgG and IgM scanned images of all HCoV peptide arrays was converted to arbitrary units (AU), pooled, background corrected, and normalized to avoid any inter-experimental variations^5,7,8^. A peptide signal was considered reactive if the intensity reading (AU) was above the threshold (mean ± 2 SD readings of random peptides, >10,000 AU for IgG and IgM analysis) (Supplementary Data 1, Supplementary Data 2). A cutoff threshold for peptide recognition was defined as mean ±2 times the standard deviation of the mean intensity value of all negative controls^11^. The regression analysis was performed to measure fold-changes on normalized and background corrected data for filtered peptides and followed by Multidimensional scaling (MDS) analysis.

**Fig. 1:**
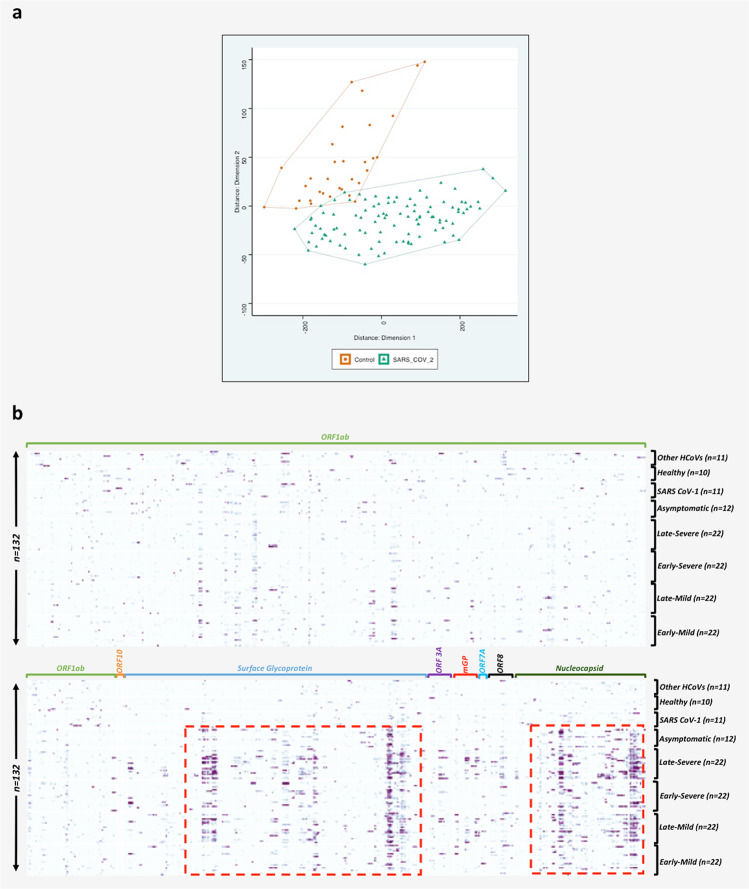
Multidimensional scaling (MDS) of differential IgG peptide signals and heatmap showing differential peptide signals throughout SARS-CoV-2 proteome. **a** Multidimensional scaling (MDS) of differential IgG peptide signals in assays of sera from subjects with a history of infection with SARS-CoV-2 (*n* = 100) or without historical exposure to SARS-CoV-2 (controls) (*n* = 32). Based on MDS analysis, samples with exposure of SARS-CoV-2 samples (green) versus controls (red) clustered into two separate groups. **b** Proteome-wide linear epitope mapping of SARS-CoV-2-specific IgG antibodies by a HCoV peptide array. *X* axis represents 981 peptides from SARS-CoV-2 proteins, *Y* axis represents 132 samples tested using the HCoV peptide array. Heatmap is plotted with normalized values of individual peptide intensity in AU for each of the 132 plasma samples. Panel grids show highly reactive areas in S and N proteins. *ORF1ab protein is large so divided partly in lower panel owing to larger size.

For IgG analysis, the >10,000 AU filtration step reduced the initial number of peptides from 172665 to 79714 for further analysis (Supplementary Data 3). A total of 37,237 peptides (18,533 from the COVID-19 group and 18,704 from the control group) were identified by regression analysis. This yielded group-specific differences (*p* < 0.05) in signal intensity. MDS analysis was performed for IgG and IgM antibodies to differentiate peptides that were immunoreactive with COVID-19 patients (Groups 1–3) versus control groups (Group 4–6). MDS analysis of these peptides confirmed separation of patients with COVID-19 (Groups 1–3) and controls (Groups 4–6) into separate clusters with minimal overlap (Fig. 1A, Supplementary Data 3). Out of 18,533 reactive 12-mer peptides associated with the COVID-19 group, 981 peptides were specific for the SARS-CoV-2 polyprotein.

The 981 SARS-CoV-2 peptides with IgG signal intensity >10,000 AU, were used to assemble a heatmap (Supplementary Data 4). The signal data was pooled from all the arrays and then we compared reactivity across different sample groups. The pooled data were normalized; background corrected, and filtered peptides with immunoreactivity above the 10,000 AU to generate the heatmap are shown in Fig. 1B (Supplementary Data 4). Immunoreactive peptides included 566 from ORF1ab, three from ORF10, 243 from surface glycoprotein (S protein), 20 from ORF3a, 20 from membrane glycoprotein (mGP), four from ORF7a, 21 from ORF8, and 104 from nucleocapsid phosphoprotein (N). Peptides from “S” and “N” proteins had higher reactive intensity (higher AU) and rate of reactivity in comparison to peptides from other proteins (Fig. 1B). Immunoreactivity was higher in COVID-19 subjects with severe versus mild disease, and in asymptomatic SARS-CoV-2-infected subjects versus subjects with mild disease. Samples from second time point collections were more reactive than first time point collections in subjects with severe, mild and asymptomatic SARS-CoV-2 infection (Fig. 1B).

The presence of a minimum of three continuous reactive peptides in samples with SARS-CoV-2 infection but non-reactive in control groups, were used to identify SARS-CoV-2 reactive IgG epitopes. Analysis of these 981 peptide sequences led to the identification of 163 epitopes (Supplementary Data 5). The 29 epitopes with the strongest and most specific reactivity with SARS-CoV-2 are shown in Table 1. Table 1 also indicates the location of each epitope on the SARS-CoV-2 proteome, its length, aa sequence, and the percentages of plasma samples that were immunoreactive in Groups 1–6. Supplementary Data 7 shows reactivity with each of these 29 epitopes for the individual plasma samples. These 29 epitopes included 11 epitopes (37.9%) in S protein (SP1-SP11), 8 (27.5%) epitopes in N protein (NP1-NP8), 6 (20.7%) epitopes in ORF1ab polyprotein (OP1–OP6), 2 (6.9%) in mGP protein (MP1 and MP2), one (3.4%) each from ORF3, and ORF8 proteins (Supplementary Fig. 1). Immunoreactivity was higher in second time point plasma samples (Supplementary Fig. 2, Supplementary Data 6) and in patients with more severe disease. In samples from patients with severe disease, 7–22 epitopes (mean of 13) were reactive in second time point samples (24.8 ± 6.8 days POD) versus 0–15 (mean of 7) epitopes in first time point samples (9.6 ± 3.5 days POD). In patients with mild disease, 3–22 epitopes (mean of eight) were reactive in second time point samples (34.7 ± 8.3 POD) versus 0–12 epitopes (mean of 4) in first time point samples (12.9 ± 5.9 POD). In asymptomatic subjects 1–9 epitopes (mean of four) were reactive at either time point (day of hospitalization and 14.5 ± 4.6 days after the hospitalization). Plasma samples from 19 of 22 patients (86%) with mild disease were reactive with at least 1 of 29 epitopes at the first time point. All 22 (100%) were reactive with at least 3 of 29 epitopes at the second time point. Plasma from 21 of 22 patients (95%) with severe disease were reactive with at least one epitope at first time point. All 22 (100%) were reactive with at least six epitopes at the second time point. All six (100%) asymptomatic cases were reactive with at least 2 of 29 epitopes in first and 3 of 29 in second timepoint collections.

**Table 1:** Characteristics of selected 29 IgG linear epitopes for detection of SARS-CoV-2 infection.

S. no.	Epitope ID.	Epitope sequence	Epitope length	AA (start)	AA (end)	Protein name (Acc. MN908947)	Severe COVID-19 Early (*N* = 22)	Severe COVID-19 Late (*N* = 22)	Mild- COVID-19 Early (*N* = 22)	Mild-COVID-19 Late (*N* = 22)	Asymptomatic COVID-19 (*N* = 12)	SARS-CoV-1 (*N* = 11)	Healthy controls (*N* = 10)	Other HCoV (*N* = 11)
1	OP1	TAYNGYLTSSSKTP	13	1484	1497	ORF1ab polyprotein (pp1ab)	5/22 (23%)	4/22 (19%)	5/22 (23%)	5/22 (23%)	0/12 (0%)	1/11 (10%)	0/10 (0%)	1/11 (10%)
2	OP2	LVPNQPYPNASFDNF	14	1914	1928		8/22 (37%)	12/22 (55%)	3/22 (14%)	3/22 (14%)	0/12 (0%)	0/11 (0%)	0/10 (0%)	0/11 (0%)
3	OP3	NRFTTTLNDFNLVAMK	15	3484	3499		1/22 (5%)	1/22 (5%)	4/22 (19%)	3/22 (14%)	0/12 (0%)	0/11 (0%)	0/10 (0%)	0/11 (0%)
4	OP4	QGLLPPKNSIDAFKLNIK	17	3826	3843		5/22 (23%)	5/22 (23%)	4/22 (19%)	6/22 (28%)	0/12 (0%)	0/11 (0%)	0/10 (0%)	2/11 (19%)
5	OP5	NTCVGSDNVTDFNAIATCDW	19	5419	5438		5/22 (23%)	13/22 (60%)	5/22 (23%)	6/22 (28%)	0/12 (0%)	0/11 (0%)	0/10 (0%)	0/11 (0%)
6	OP6	FYSYATHSDKFTDGV	14	6292	6306		7/22 (32%)	11/22 (50%)	0/22 (0%)	2/22 (10%)	0/12 (0%)	0/11 (0%)	0/10 (0%)	0/11 (0%)
7	SP1	TESNKKFLPFQQFGRDIAD	18	553	571	Surface glycoprotein	4/22 (19%)	15/22 (69%)	1/22 (5%)	9/22 (41%)	3/12 (25%)	0/11 (0%)	0/10 (0%)	0/11 (0%)
8	SP2	TDAVRDPQTLEILDITPCS	18	573	591		6/22 (28%)	17/22 (78%)	1/22 (5%)	11/22 (50%)	2/12 (17%)	0/11 (0%)	0/10 (0%)	0/11 (0%)
9	SP3	QVAVLYQDVNCTEVPVAIHADQLTPTW	26	607	633		0/22 (0%)	9/22 (41%)	2/22 (10%)	4/22 (19%)	0/12 (0%)	0/11 (0%)	0/10 (0%)	0/11 (0%)
10	SP4	CASYQTQTNSPRRARSV	16	671	687		2/22 (10%)	4/22 (19%)	0/22 (0%)	1/22 (5%)	2/12 (17%)	0/11 (0%)	0/10 (0%)	0/11 (0%)
11	SP5	ARSVASQSIIAYTMSLGAENSVA	22	684	706		2/22 (10%)	6/22 (28%)	4/22 (19%)	4/22 (19%)	2/12 (17%)	0/11 (0%)	0/10 (0%)	0/11 (0%)
12	SP6	RALTGIAVEQDKNTQEVFAQV	20	765	785		11/22 (50%)	14/22 (64%)	2/22 (10%)	5/22 (23%)	2/12 (17%)	0/11 (0%)	0/10 (0%)	0/11 (0%)
13	SP7	FAQVKQIYKTPPIKDFGGFNF	20	782	802		5/22 (23%)	8/22 (37%)	1/22 (5%)	3/22 (14%)	2/12 (17%)	0/11 (0%)	0/10 (0%)	0/11 (0%)
14	SP8	NFSQILPDPSKPSKRS	15	801	816		1/22 (5%)	4/22 (19%)	0/22 (0%)	4/22 (19%)	2/12 (17%)	0/11 (0%)	0/10 (0%)	0/11 (0%)
15	SP9	KRSFIEDLLFNKVT	13	814	827		5/22 (23%)	8/22 (37%)	4/22 (19%)	5/22 (23%)	1/12 (9%)	0/11 (0%)	0/10 (0%)	0/11 (0%)
16	SP10	PELDSFKEELDKYFKNHTSPDVD	22	1143	1165		15/22 (69%)	21/22 (96%)	9/22 (41%)	18/22 (82%)	8/12 (67%)	1/11 (10%)	0/10 (0%)	1/11 (10%)
17	SP11	SPDVDLGDISGINASVV	16	1161	1177		10/22 (46%)	20/22 (91%)	6/22 (28%)	13/22 (60%)	0/12 (0%)	1/11 (10%)	2/10 (20%)	1/11 (10%)
18	ORF3-1	EHDYQIGGYTEKWESGVKDCVVL	22	181	203	ORF3a protein	4/22 (19%)	7/22 (32%)	1/22 (5%)	2/22 (10%)	0/12 (0%)	0/11 (0%)	0/10 (0%)	0/11 (0%)
19	MP1	CDIKDLPKEITVATS	14	159	173	Membrane glycoprotein	0/22 (0%)	8/22 (37%)	0/22 (0%)	6/22 (28%)	0/12 (0%)	0/11 (0%)	0/10 (0%)	0/11 (0%)
20	MP2	QRVAGDSGFAAYSRY	14	185	199		0/22 (0%)	10/22 (46%)	0/22 (0%)	4/22 (19%)	0/12 (0%)	0/11 (0%)	0/10 (0%)	0/11 (0%)
21	ORF8-1	GSKSPIQYIDIGNYTVSCLPF	20	66	86	ORF8 protein	3/22 (14%)	12/22 (55%)	4/22 (19%)	3/22 (14%)	0/12 (0%)	0/11 (0%)	0/10 (0%)	1/11 (10%)
22	NP1	ALLLLDRLNQLESKMSG	16	220	236	Nucleocapsid phosphoprotein	3/22 (14%)	5/22 (23%)	2/22 (10%)	5/22 (23%)	2/12 (17%)	0/11 (0%)	0/10 (0%)	0/11 (0%)
23	NP2	NQLESKMSGKGQQQQGQTVTKKSA	23	228	251		12/22 (55%)	18/22 (82%)	8/22 (37%)	15/22 (69%)	7/12 (59%)	0/11 (0%)	0/10 (0%)	0/11 (0%)
24	NP3	QTVTKKSAAEASKKPRQK	17	244	261		2/22 (10%)	7/22 (32%)	1/22 (5%)	4/22 (19%)	2/12 (17%)	0/11 (0%)	0/10 (0%)	0/11 (0%)
25	NP4	RGPEQTQGNFGDQELI	15	277	292		6/22 (28%)	3/22 (14%)	2/22 (10%)	3/22 (14%)	2/12 (17%)	0/11 (0%)	0/10 (0%)	0/11 (0%)
26	NP5	AYKTFPPTEPKKDKKKK	16	359	375		2/22 (10%)	5/22 (23%)	0/22 (0%)	2/22 (10%)	2/12 (17%)	0/11 (0%)	0/10 (0%)	0/11 (0%)
27	NP6	KDKKKKADETQALPQR	15	370	385		3/22 (14%)	7/22 (32%)	0/22 (0%)	5/22 (23%)	3/12 (25%)	0/11 (0%)	0/10 (0%)	0/11 (0%)
28	NP7	ADETQALPQRQKKQQTVTLLPAADL	24	376	400		4/22 (19%)	17/22 (78%)	4/22 (19%)	9/22 (41%)	2/12 (17%)	0/11 (0%)	0/10 (0%)	0/11 (0%)
29	NP8	TLLPAADLDDFSKQLQQSMSSADS	23	393	416		14/22 (64%)	22/22 (100%)	6/22 (28%)	18/22 (82%)	7/12 (59%)	1/11 (10%)	0/10 (0%)	0/11 (0%)

Sequences (aa), length, aa location in protein is based on proteome of Severe acute respiratory syndrome coronavirus 2 isolate Wuhan-Hu-1 (accession no. MN908947).

Plasma from none of the asymptomatic cases was reactive with any ORF1ab, ORF3, ORF8, or mGP epitope (Supplementary Table 4). Plasma from asymptomatic cases were reactive only with S and N epitopes. Plasma collected at both time points from asymptomatic case AS1 was reactive with five epitopes in the S protein (SP1, SP2, SP4, SP5, and SP10) and three epitopes in the N protein (NP2, NP7, and NP8). Case AS1 plasma was reactive with SP9 at only first time point. Plasma from asymptomatic case AS2 was reactive with three epitopes in the S protein (SP7, SP8, and SP10) and five epitopes in the N protein (NP2, NP3, NP4, NP5, and NP8) at both time points. Plasma from asymptomatic cases AS1, AS2, and AS4 were reactive with SP10 and NP8 epitopes at both time points. Plasma from AS3 was reactive with SP10 at both time points and with NP8 only at the second time point. Plasma from AS5 and AS6 were immunoreactive with the NP1 epitope and the epitope NP6, respectively, at both time points. Plasma from mild case M7 was reactive only with the SP10 epitope at first time point but was reactive to 22/29 epitopes at second time point of collection. All mild and severe COVID-19 cases were reactive to a greater number of epitopes at second time points versus the first time point (Supplementary Data 7).

**Fig. 2:**
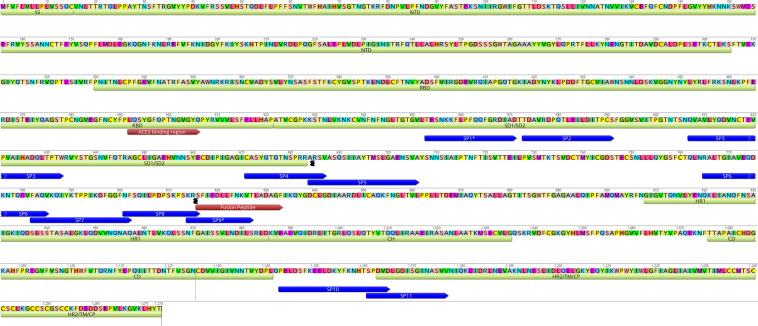
Mapping of eleven spike epitopes identified in this study on the surface glycoprotein of SARS-CoV-2 [GenBank: QHD43416.1]. Location of Spike epitopes (SP1-SP11) is shown that in dark blue arrows. Primary structure domains are colored by green bars: SS, signal sequence; S2′, S2′ protease cleavage site; FP, fusion peptide; HR1, heptad repeat 1; CH, central helix; CD, connector domain; HR2, heptad repeat 2; TM, transmembrane domain; CT, cytoplasmic tail. “{” symbols indicate protease cleavage sites. Brown arrows denote ACE2-binding region and fusion peptide regions. *Epitopes with neutralizing antibody binding potential (SP1*, and SP9*).

The 29 immunoreactive epitopes were mapped to the proteome of SARS-CoV-2 (acc. number MN908947) (Supplementary Fig. 1). ORF1ab epitopes (OP1–OP6) were dispersed throughout the protein. Eleven linear epitopes from “S” protein (SP1-SP11) mapped outside of the RBD (Receptor Binding Domain) (Fig. 2). Four (SP1-SP4) epitopes were located in the SD1/SD2. Four (SP5-SP8) epitopes were located between SD1/SD2 and fusion peptide region. The SP9 epitope overlapped the fusion peptide. The SP10 epitope is located between CD (Connector Domain) region and HR2 (Heptad Repeat 2). The SP11 epitope was located at the beginning of the HR2 region of the S2 subunit of the surface glycoprotein (Fig. 2). The SP1 and SP9 peptides identified in this study were recently reported as potentially linked to neutralization^12^.

Ten of eleven spike epitopes (SP1–SP10) were located in regions of high immunoreactivity reported in a recent pre-print from Li et al.^13^. The SP10 epitope was the most reactive spike epitope in subjects with severe (69% first time point, 96% second timepoint), mild (41% first timepoint, 82% second timepoint), and asymptomatic SARS-CoV-2 (67% either timepoint) infections. The NP2 and NP8 epitopes were the most reactive N protein epitopes. In severe disease, NP2 reactivity was found in 55% of subjects at the first timepoint, and 82% at the second timepoint. NP8 reactivity was found in 64% of subjects at first timepoint, and 100% at second timepoint. In mild disease, NP2 reactivity was found in 37% of subjects at the first timepoint, and 69% at the second timepoint. NP8 reactivity was found in 28% of subjects at the first timepoint, and 82% at the second timepoint. In asymptomatic cases, reactivity was 59% for either NP2 or NP8. In severe disease SP11 reactivity was found in 46% of subjects at the first time point, and 91% at the second time point. In mild disease SP11 reactivity was found in 28% of subjects at the first time point, and 60% at the second time point. No asymptomatic cases were reactive with SP11. Epitopes for ORF1ab (OP1–OP6) showed reactivity in cases with mild and severe disease at both timepoints but not in asymptomatic infections. Only second timepoint samples from severe and mild diseases were reactive to epitopes for mGP, MP1, and MP2 (37 and 26% for severe disease and 28 and 19% for mild disease).

IgM analysis revealed one linear epitope in the membrane glycoprotein (MADSNGTITVEELKKLLEQWN), that was reactive in 4/22 (19%) COVID-19 patients with mild disease and 8/22 (37%) patients with severe disease at late time point collection (Supplementary Fig. 3, Supplementary Table 5, Supplementary Data 8).

Through use of a limited number of plasma samples from subjects with known exposure to other HCoV we also identified IgG epitopes in HKU1 (*n* = 15), NL63 (*n* = 10), OC43 (*n* = 14), 229E (*n* = 5), and SARS-CoV-1 (*n* = 9) (Supplementary Table 6). Whether these epitopes will have diagnostic utility cannot be determined due to the small sample size. We were unable to test for MERS-specific epitopes due to lack of cognate sera. More than 90% of samples from patients exposed to SARS-CoV-2 and controls showed a wide range of reactivity to epitopes from seasonal coronaviruses (HKU1, NL63, OC43, and 229E).

In summary, we used the HCoV peptide array and plasma from 50 patients with asymptomatic, mild, or severe SARS-CoV-2 infection to identify immunoreactive IgG epitopes for SARS-CoV-2. Immunoreactivity profiles differed with severity of illness and over the time course of infection. Two subjects with a history of SARS-CoV-1 infection had reactivity to two of 29 IgG SARS-CoV-2 epitopes. Their plasma was collected in 2004 or 2005; thus, this presumably reflects cross-reactivity due to proteome homology^2,3^. Two healthy controls with immunoreactivity to SP11 (one of 29 epitopes of SARS-CoV-2) may have had an asymptomatic infection with either SARS-CoV-1 or SARS-CoV-2^14,15^.

The HCoV array platform is too complex and expensive for routine clinical microbiology. However, the peptides defined here can be transferred to a wide range of platforms including microarrays, enzyme-linked immunosorbent assay, radioimmunoassay, lateral flow, western blot, and bead-based assays, where they may facilitate diagnostics, epidemiology, and vaccinology.

## Methods

### HCoV peptide array design

We employed a programmable peptide microarray that can accommodate up to three million distinct linear peptides on a 75 mm × 26 mm slide. The array can also be divided into 12 subarrays, each containing ~172,000 12-mer peptides (Nimble Therapeutics Inc, WI, USA). To enable differential detection of antibodies specific for SARS-CoV-2 infections, we created a database comprising the proteomes of seven HCoVs: SARS-CoV-2, SARS, MERS, NL63, OC43, 229E, and HKU1 (Supplementary Table 1). We also included two bat coronavirus proteomes similar to SARS-CoV-2^16^. In all, 1000 randomly selected 12 aa long scrambled peptides were added for background correction and nonspecific binding of peptides. For each virus selected, we downloaded all available protein sequences available before January 2020 from the NCBI and Virus Pathogen Database and Analysis Resource protein databases. We then created a peptide database comprising overlapping 12-mer peptides that tiled the whole proteome of each of these agents with 11 amino acid (aa) overlap in a sliding window pattern^4–7^. These viral sequences resulted a total of unique 172,665 peptides. Redundant peptides were excluded prior to synthesis. The individual peptides in the library were printed in random positions on the peptide array to minimize the impact of locational bias.

### Samples and experimental design

The study was approved by the Medical Ethical Committee of Sun Yat-Sen University (approval number 2020–060). An informed and written consent was obtained from all patients. A total 132 plasma samples were tested and analyzed using HCoV peptide arrays (Supplementary Table 2). Samples were divided into six groups: group 1] COVID-19 patients with mild disease (*n* = 22); group 2] COVID-19 patients with severe disease (*n* = 22); group 3] patients with SARS-CoV-2 infections but no-symptoms (asymptomatic COVID-19) (*n* = 6); group 4] SARS-CoV-1 (2003) IgG-positive cases (*n* = 11); group 5] other banked HCoV IgG-positive controls (*n* = 11) (Supplementary Table 3); and group 6] healthy controls (*n* = 10). The average age was 44.0 ± 16.73 years for the mild-COVID-19 group, 60.1 ± 12.37 years for the severe COVID-19 group, and 43.5 ± 15.08 years for the asymptomatic COVID-19 group. The asymptomatic group subjects were family members or close contacts of the mild or severely ill patients. The average age for SARS-CoV-1 IgG-positive control group was 24.4 ± 5.0 years. The average age for healthy control group was 46.3 ± 7.3 years. Other HCoV controls included banked samples for which age and sex information is not available. Plasma samples were collected at two different time points, a minimum of two weeks apart, from group COVID-19 patients (Groups 1, 2, and 3). The first time point (early) was collected at 12.9 ± 5.9 POD for the mild disease group, and at 9.6 ± 3.5 days POD for the severe disease group. The second time point (late was collected at 34.7 ± 8.3 days POD for the mild disease group, and at 24.8 ± 6.8 days POD for the severe disease group. For asymptomatic group, the first time point was collected on the day of hospitalization; the second time point was collected at 14.5 ± 4.6 days after the day of hospitalization. Non-COVID-19 samples from control groups (Group 4, Group 5, and Group 6), were from adults without any evidence or history of infection with SARS-CoV-2. All COVID-19 patients were tested positive for SARS-CoV-2 RNA in respiratory specimens using the China FDA approved Novel Coronavirus (2019-nCoV) Real-Time RT-PCR kit from LifeRiver Ltd. (Catalog #: RR-0479-02) real-time RT-PCR^17^. The diagnosis of COVID-19 pneumonia, and severity criteria were assessed at Guangdong CDC based on the New Coronavirus Pneumonia Prevention and Control Program (6th edition) published by the National Health Commission of China^17^. Other clinical information, comorbidities, symptoms, and treatment for COVID-19 for mild, severe, and asymptomatic cases are presented in Supplementary Table 2.

### HCoV peptide array synthesis, sample binding, and processing

Microarrays were synthesized with a Nimble Therapeutics Maskless Array Synthesizer by light-directed solid-phase peptide synthesis using an amino-functionalized support (Greiner Bio-One) coupled with a 6-aminohexanoic acid linker and amino acid derivatives carrying a photosensitive 2-(2-nitrophenyl) propyloxycarbonyl (NPPOC) protection group (Orgentis Chemicals). Amino acids (final concentration 20 mM) were pre-mixed for 10 min in *N,N*-Dimethylformamide (DMF, Sigma Aldrich) with *N,N,N’,N’*-Tetramethyl-O-(1H-benzotriazol-1-yl) uronium-hexafluorophosphate (HBTU, Protein Technologies, Inc.; final concentration 20 mM) as an activator, 6-Chloro-1-hydroxybenzotriazole (6-Cl-HOBt, Protein Technologies, Inc.; final concentration 20 mM) to suppress racemization, and *N,N*-Diisopropylethylamine (DIPEA, Sigma Aldrich; final concentration 31 mM) as base. Activated amino acids were then coupled to the array surface for 3 min. Following each coupling step, the microarray was washed with *N*-methyl-2-pyrrolidone (VWR International), and site-specific cleavage of the NPPOC protection group was accomplished by irradiation of an image created by a Digital Micro-Mirror Device (Texas Instruments), projecting 365 nm wavelength light. Coupling cycles were repeated to synthesize the full in silico-generated peptide library.

We used 11 arrays to test 132 plasma samples. Before loading, plasma samples were heat-inactivated at 56 °C for 30 min. Plasma samples were diluted (1:50) with binding buffer (0.1 M Tris-Cl, 1% alkali soluble casein, 0.05% Tween-20, and water). The peptide arrays were incubated overnight at 4 °C on a flat surface with individual sample/subarray. Overnight sample incubation was followed by three 10-minute washes with 1× TBST (0.05% Tween-20) at room temperature (RT). Secondary antibodies IgG (cat no. 109-605-098, Alexa Fluor 647-AffiniPure Goat Anti- Human IgG, Fcy fragment specific, Jackson ImmunoResearch Labs) and IgM (cat no. 109-165-129, Cy™3 AffiniPure Goat Anti-Human IgM, Jackson ImmunoResearch Labs) were diluted in 1× PBS at a concentration of 0.1 µg/ml, and arrays were incubated in Plastic Coplin Jar (cat no. S90130, Fisher Scientific) for 3 h at RT with gentle shaking. Secondary antibody incubation was also followed by three 10-minute washes with 1× TBST at RT. After a final wash, the arrays were dried and scanned on a microarray scanner at 2-μm resolution, with an excitation wavelength of 635 nm (IgG) and 532 nm (IgM). Scanned array images were analyzed with proprietary Nimble Therapeutics software to extract fluorescence intensity values for each peptide. The fluorescent signals were converted into AU intensity plots ranging minimum to maximum intensity 0–65,000 AU.

### Quality and reproducibility of the array data

In the array synthesis process, Nimble Therapeutics uses a quality control step that builds thousands of peptides with known binding epitopes to streptavidin, which have been confirmed through SPR and crystallization method^18^. Each subarray on the entire synthesized array slide contains these quality control peptides in addition to the customized targeted experimental peptides. Additionally, every array slide is QC analyzed and the signal AU from these control peptides for each slide is correlated to the banked data from previous QC analyses. Arrays that do not meet standard cutoff thresholds are deemed as failed and removed from the further experimental process. Random non-adjacent tiling of overlapping peptides also enhances confidence in the probe quality and supports the reactivity of an individual epitope by having multiple, overlapping peptides per epitope. In our previous studies, we have shown epitope reactivity of 12 aa overlapping peptides with an 11 aa overlap (single aa tiling). In Supplementary Fig. 4 we have illustrated examples of how randomly tiled peptide sequences generate >10,000 AU signal and formed SP10 and NP8 reactive epitopes in plasma from a severe COVID-19 case and has <10,000 AU reactivity in plasma sample from healthy control subject. The reproducibility of data and inter-array reproducibility analysis of peptide arrays has been reported in previous studies by testing technical replicates on two separate microarrays. The two technical replicates for the target epitopes showed almost identical results with distinct epitopes^5,7,19^.

All analysis similar to IgG was also performed to generate data from IgM reactive peptides. For IgM analysis, >10,000 AU filtration step reduced the initial number of peptides from 172665 to 24728 for further analysis. A total of 10,816 peptides (7144 from the COVID-19 group and 3672 from the control group) peptides yielded group-specific differences (*p* < 0.05) in signal intensity. MDS efforts did not separate samples collected from patients with COVID-19 and controls (Supplementary Fig. 3). However, the presence of three continuous peptides that were reactive in samples from SARS-CoV-2-infected subjects (irrespective of disease status) but not in control groups, allowed the identification of 16 SARS-CoV-2-specific IgM epitopes (Supplementary Table 5).

### Statistics and reproducibility

Reactivity values for all 132 samples were pooled together and peptides showing >10,000 AU for any samples, were retained for further analysis. Regression analysis, fold-changes, and standard errors were estimated by fitting a linear model for signal intensities generated by each peptide, applying empirical Bayesian smoothing to the standard errors, and then determining those peptides that yielded statistically significant signal by contrasting linear models for each peptide between SARS-CoV-2 and Control samples at a significance value of <0.05^20^. edgeR package in R was used for this purpose. It implements quantile-adjusted conditional maximum-likelihood method for estimating dispersions followed by fitting negative binomial generalized linear models. This is followed by a quasi-likelihood F-test to determine those peptides that yielded statistically significant signal by contrasting linear models for each peptide between SARS-CoV-2 and Control samples. MDS plots (Fig. 1A, Supplementary Fig. 3) shows the discriminative ability of these peptides. These peptides were then used to reassemble longer epitope sequences (Table 1, Supplementary Table 5, Supplementary Data 5). The normalization, background correction was performed using preprocessCore R package^21^ and statistical comparison of peptide microarray intensities between groups were performed using the edgeR package^22^.

Reactivity values for all 132 samples were pooled together and peptides showing >10,000 AU for any samples, were retained for further analysis. Regression analysis, fold-changes, and standard errors were estimated by fitting a linear model for signal intensities generated by each peptide, applying empirical Bayesian smoothing to the standard errors, and then determining those peptides that yielded statistically significant signal by contrasting linear models for each peptide between SARS-CoV2 and control samples at a significance value of <0.05^20^. The normalization, background correction was performed using preprocessCore R package^21^ and statistical comparison of peptide microarray intensities between groups were performed using the edgeR package^22^. The analysis was performed to differentiate peptides that were immunoreactive with COVID-19 patients (groups 1, 2, and 3) and versus control groups (groups 4, 5, and 6) samples. MDS plots were generated using signal data for these significant peptides. The code for reassembly and plots was prepared using Rstudio v 1.2.5019^23^. The plots were generated using ggplot2 package^24^. A custom color-blind friendly color pallete was used to make the plots. Alignment of reactive epitopes on SARS-CoV-2 proteome was performed using Geneious version 10.0.9.

### Reporting summary

Further information on research design is available in the Nature Research Reporting Summary linked to this article.

## Supplementary information

Supplementary Information

Description of Supplementary Files

Supplementary Data 1

Supplementary Data 2

Supplementary Data 3

Supplementary Data 4

Supplementary Data 5

Supplementary Data 6

Supplementary Data 7

Supplementary Data 8

Reporting Summary

## Data Availability

All data generated during this study are included in this published article and its supplementary information files. The supporting data analysis code is available on Github (https://github.com/ciibioinformatics/COVID19_publication). The source data underlying plots shown in figures are provided in Supplementary Data 1–8. Additional data inquiries can be made to the corresponding authors of this manuscript.
