# Author Correction: Immunoreactive peptide maps of SARS-CoV-2 and other human coronaviruses

**DOI:** 10.1038/s42003-021-01866-z

**Published:** 2021-03-09

**Authors:** Nischay Mishra, Xi Huang, Shreyas Joshi, Cheng Guo, James Ng, Riddhi Thakkar, Yongjian Wu, Xin Dong, Qianlin Li, Richard S. Pinapati, Eric Sullivan, Adrian Caciula, Rafal Tokarz, Thomas Briese, Jiahai Lu, W. Ian Lipkin

**Affiliations:** 1 Center for Infection and Immunity, Mailman School of Public Health, Columbia University, New York, NY USA; 2 Sun Yat-sen University, Guangzhou, Guangdong Province China; 3 Center for Infection and Immunity, Fifth Affiliated Hospital, Sun Yat-sen University, Zhuhai, Guangdong Province China; 4 School of Public Health, Sun Yat-sen University, Guangzhou, Guangdong Province China; 5 Nimble Therapeutics Inc., Madison, WI USA

**Keywords:** Infectious-disease diagnostics, SARS-CoV-2, Infection, Viral infection

Correction to: *Communications Biology* 10.1038/s42003-021-01743-9, published online 12 February 2021.

The original version of the Article contained an error in Fig. 1b, in which the sample sizes for the following labels were incorrectly given as *n* = 10; the correct sample sizes are *n* = 22 for each label, respectively: *Late-Severe, Early-Severe, Late-Mild*, and *Early-Mild*. The correct version of Fig. 1 is which replaces the previous incorrect version. The error has been corrected in the HTML and PDF versions of the Article.

