# Supplementary material for: Immunoreactive peptide maps of SARS-CoV-2
Source: Commun Biol. 2021 Feb 12;4:225. doi: 10.1038/s42003-021-01743-9 (PMC7881038; doi:10.1038/s42003-021-01743-9)
Supplement: Supplementary file 1 — Supplementary Information [file 42003_2021_1743_MOESM1_ESM.pdf]

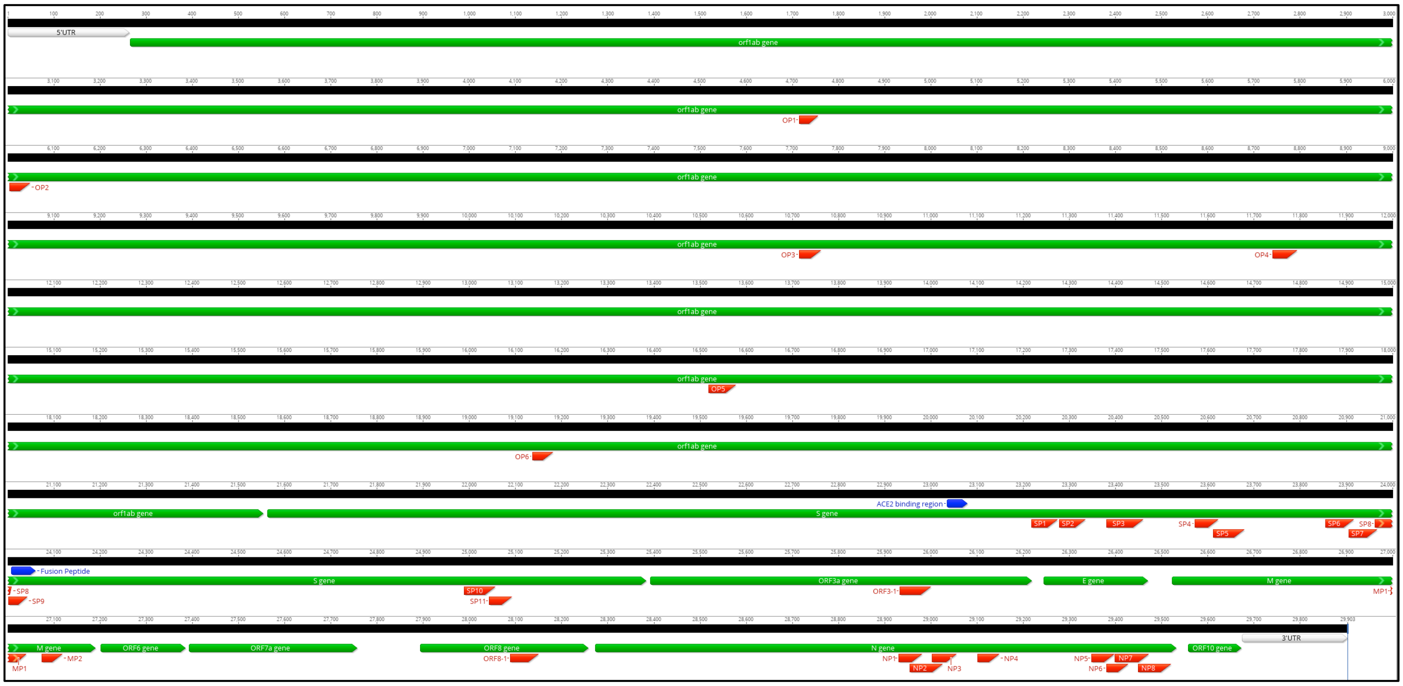

Supplementary figure 1. Notation of twenty-nine immunogenic B cell IgG epitopes on the proteome of SARS-CoV-2 (Accession number MN908947)

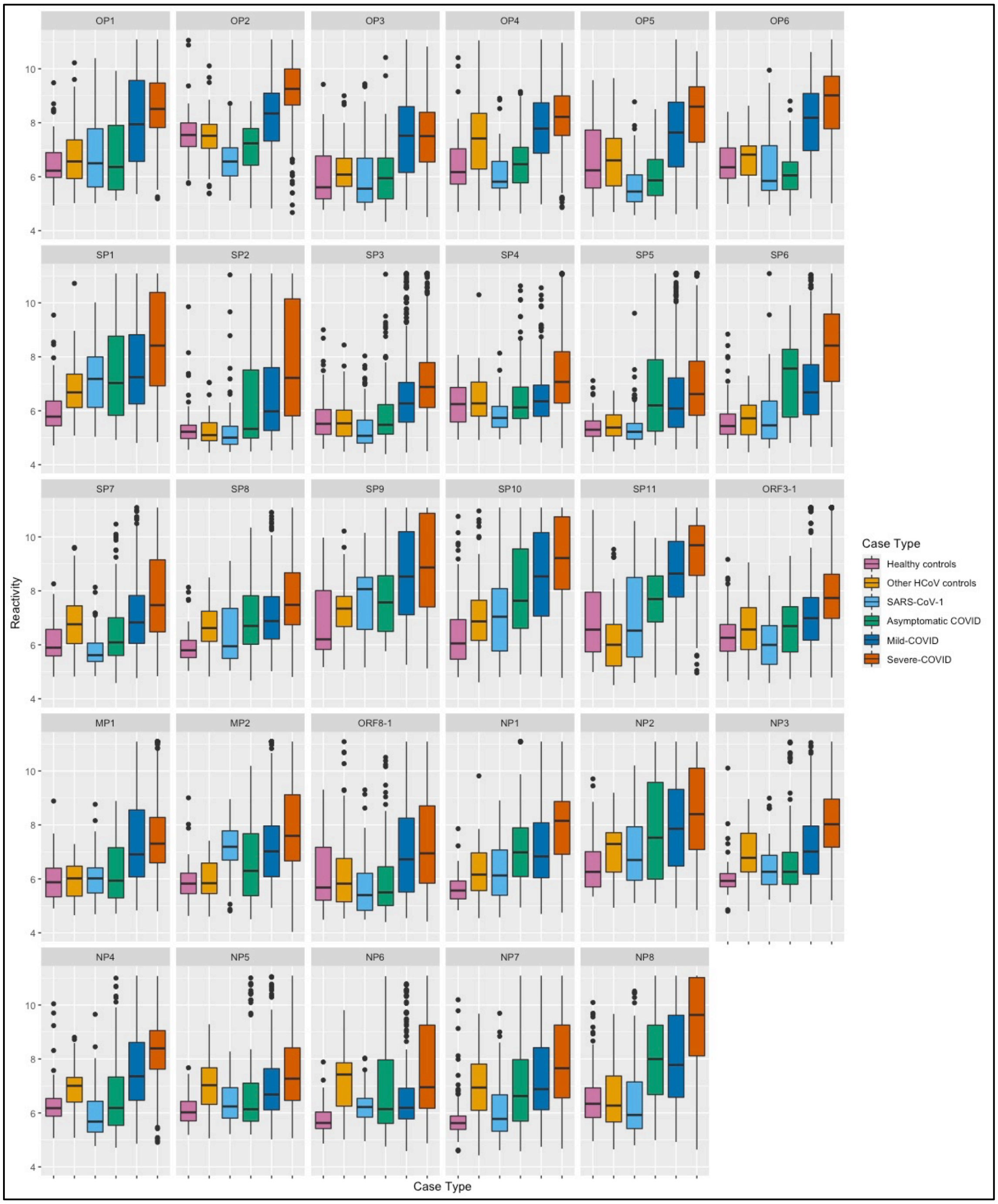

Supplementary figure 2. Reactivity intensities of each peptide from 29 immunogenic linear IgG epitopes are plotted on log scale (y-axis). Reactivity to selected peptides is plotted for corresponding groups shown in different colors (x-axis).

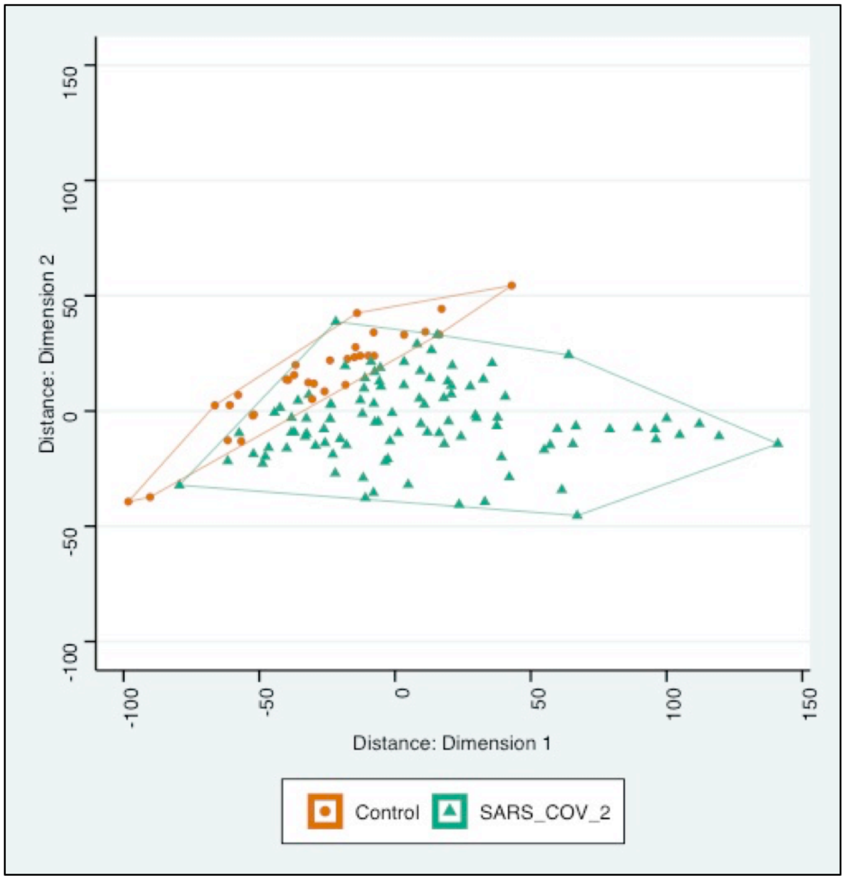

Supplementary figure 3. Multidimensional scaling (MDS) of differential IgM peptide signals in assays of sera from subjects with a history of infection with SARS-CoV-2 (n=100) or without historical exposure to SARS-CoV-2 (controls) (n=32). Based on MDS analysis, samples with exposure of SARS-CoV-2 samples (green) versus controls (red) clustered in overlapping groups.

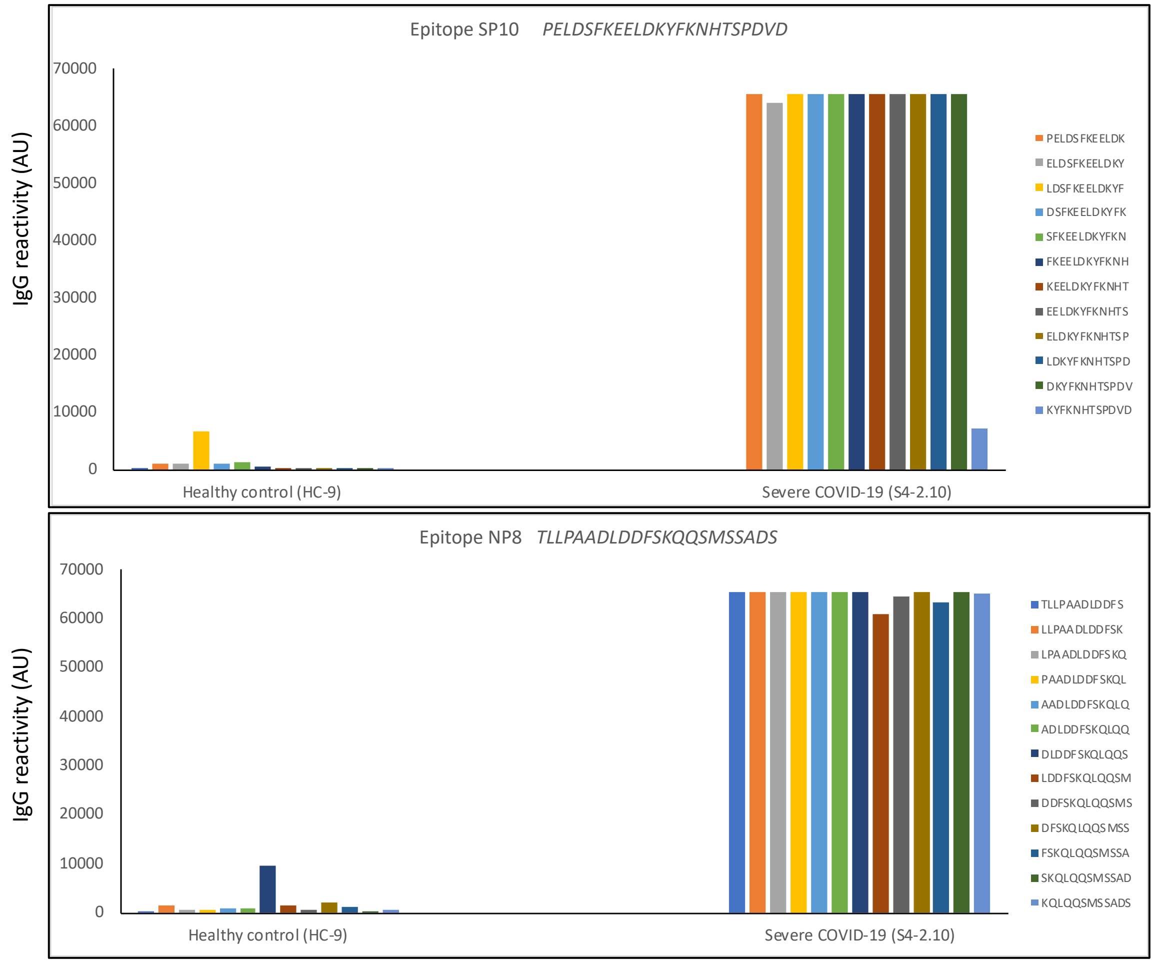

Supplementary figure 4. Illustration of overlapping 12 aa peptides reactivity and formation of SP10 and NP8 epitopes. Reactivity was measured in arbitrary units (X axis) and compared between in a severely ill COVID-19 patient versus healthy control sample (Y axis).

	Virus database	No. of protein sequences	Total redundant "12-aa" peptides	Total unique "12-aa" peptides
1	*SARS-CoV-2-MN908947-CDS*	10	9591	9591
2	*Human-229E-CoV-PDB*	662	576999	15756
3	*Human-HKU1-CoV-PDB*	721	554447	16390
4	*Human-NC63-CoV-PDB*	1320	1000287	16392
5	*Human-OC43-CoV-PDB*	2667	2679272	29635
6	*MERS-CoV-PDB*	7253	8604059	54188
7	*SARS-CoV-PDB*	1996	1484140	29791
8	*Human-CoV-variants-VIPR-PDB*	12131	9880717	101145
9	*Bat-CoV-ZC45*	11	9678	9678
10	*Bat-CoV-ZXC21*	11	9655	9655
11	*Redundant-non-specific-peptides*	NA	NA	1000
Total number of unique and redundant peptides			24779921	283630
**Total peptides in HCOV-SeroChip**			**172665**	

Supplementary Table 1. Design and characteristics of HCoV-peptide array

	Asymptomatic (n=6)	Non-severe (n=22)	Severe (n=22)	SARS-CoV-1 (n=11)	Healthy (n=10)	Other HCoV (n=11)
Gender (M/F)	2/4	13/9	14/8	1/10	5/5	--
Age (years)	43.5±15.08	44.0±16.73	60.1±12.37	24.4±5.0	46.3±7.3	--
Time of collection (post onset of symptoms days, POD)						
First collection	day of hospitalisation	12.9±5.9	9.6±3.5	--	--	--
Second collection	14.5±4.6	34.7±8.3	24.8±6.8	--	--	--
Symptoms						
Cough	0(0%)	19(86%)	15(68%)	--	--	--
Fever	1(17%)	16(73%)	19(86%)	--	--	--
Dyspnea	0(0%)	2(9%)	5(23%)	--	--	--
Snivel	0(0%)	3(14%)	1(5%)	--	--	--
Sore throat	0(0%)	4(18%)	5(23%)	--	--	--
Diarrhea/Vomit/Stomach ache	0(0%)	7(32%)	8(36%)	--	--	--
Underlying diseases						
Cancer	1(17%)	2(9%)	0(0%)	--	--	--
Thyroid disease	1(17%)	0(0%)	2(9%)	--	--	--
Diabetes	0(0%)	2(9%)	3(14%)	--	--	--
Hypertension	0(0%)	5(23%)	6(27%)	--	--	--
Arthritis	0(0%)	1(5%)	0(0%)	--	--	--
Kidney disease	0(0%)	1(5%)	1(5%)	--	--	--
Appendicitis History	0(0%)	1(5%)	1(5%)	--	--	--
Gastritis history	0(0%)	0(0%)	1(5%)	--	--	--
Spinal diseases	0(0%)	0(0%)	2(9%)	--	--	--
Bronchiectasis	0(0%)	0(0%)	1(5%)	--	--	--
Skin disease	0(0%)	0(0%)	1(5%)	--	--	--
Treatment						
Antibiotic	3(50%)	17(77%)	22(100%)	--	--	--
Antivirus	5(83%)	19(86%)	21(95%)	--	--	--
Interferon	0(0%)	1(5%)	3(14%)	--	--	--
TCM	1(17%)	7(32%)	6(27%)	--	--	--
Globulin	0(0%)	11(50%)	21(95%)	--	--	--
Chloroquine	5(83%)	16(73%)	9(41%)	--	--	--

Supplementary Table 2. Clinical characteristics of patients by groups with SARS-CoV-2 infection and controls.

Sample-ID	229E-CoV	NL63-CoV	OC4-3 CoV	HKU1-CoV
PPA-0166	++++	N	I	++
PPA-0178	+++	+	I	I
PPA-0172	++++	I	+	++++
PPA-0176	++++	++++	++++	++++
PPA-0165	N	N	N	*I*
PPA-0170	*I*	*I*	N	N
PPA-0177	N	*I*	N	N
PPA-0168	N	+	I	++++
PPA-0179	+	++	N	++++
PPA-0173	N	I	+++	++++
PPA-0169	N	++++	+++	N

NA	Data not available
I	*Indeterminate*
N	*Negative*
++	*Positive*
+++	*Strong Positive*
++++	*Very Strong Positive*

Supplementary Table 3. IgG Immunoreactivity of other HCoV controls using S protein based specific ELISAs for four seasonal human coronaviruses (OC43, 229E, HKU1 and NL63).

Epitope ID	Epitope name	Epitope sequence	Group 1-Severe cases of COVID-19 (n=22)			Group 2-Mild cases of COVID-19 (n=22)			Group 3-Aysmptomatic cases of		
			Reactive in first time point collection	Reactive in second time point collection	Reactive in both time point collections	Reactive in first time point collection	Reactive in second time point collection	Reactive in both time point collections	Reactive in first time point collection	Reactive in second time point collection	Reactive in both time point collections
1	OP1	TAYNGYLTSSSKTP	1/22 (4.5%)	0/22(0%)	4/22(18.2%)	1/22(4.5%)	1/22(4.5%)	4/22(18.2%)	0/6(0%)	0/6(0%)	0/6(0%)
2	OP2	LVPNQPYPNASFDNF	0/22(0%)	4/22(18.2%)	8/22(36.4%)	1/22(4.5%)	1/22(4.5%)	2/22 (9.1%)	0/6(0%)	0/6(0%)	0/6(0%)
3	OP3	NRFTTTLNDFNLVAMK	0/22(0%)	0/22(0%)	1/22 (4.5%)	1/22(4.5%)	0/22(0%)	3/22(13.6%)	0/6(0%)	0/6(0%)	0/6(0%)
4	OP4	QGLLPPKNSIDAFKLNIK	2/22 (9.1%)	2/22 (9.1%)	3/22(13.6%)	0/22(0%)	2/22 (9.1%)	4/22(18.2%)	0/6(0%)	0/6(0%)	0/6(0%)
5	OP5	NTCVGSDNVTDFNAIATCDW	0/22(0%)	8/22(36.4%)	5/22(22.7%)	2/22 (9.1%)	3/22(13.6%)	3/22(13.6%)	0/6(0%)	0/6(0%)	0/6(0%)
6	OP6	FYSYATHSDKFTDGV	2/22 (9.1%)	6/22 (27.3%)	5/22(22.7%)	0/22(0%)	2/22 (9.1%)	0/22(0%)	0/6(0%)	0/6(0%)	0/6(0%)
7	ORF3-1	EHDYQIGGYTEKWESGVKDCVVL	1/22 (4.5%)	2/22 (9.1%)	4/22(18.2%)	0/22(0%)	1/22 (4.5%)	1/22 (4.5%)	0/6(0%)	0/6(0%)	0/6(0%)
8	ORF8-1	GSKSPIQYIDIGNYTVSCLPF	1/22 (4.5%)	5/22(22.7%)	4/22(18.2%)	2/22 (9.1%)	1/22 (4.5%)	2/22 (9.1%)	0/6(0%)	0/6(0%)	0/6(0%)
9	SP1	TESNKKFLPFQQFGRDIAD	0/22(0%)	13/22(59.1%)	3/22(13.6%)	0/22(0%)	8/22(36.4%)	2/22 (9.1%)	0/6(0%)	1/6(16.7%)	1/6(16.7%)
10	SP2	TDAVRDPQTLEILDITPCS	1/22 (4.5%)	14/22(63.6%)	4/22(18.2%)	0/22(0%)	10/22(45.5%)	2/22 (9.1%)	0/6(0%)	0/6(0%)	1/6(16.7%)
11	SP3	QVAVLYQDVNCTEVPVAIHADQLTPTW	0/22(0%)	9/22(40.9%)	0/22(0%)	0/22(0%)	2/22 (9.1%)	2/22 (9.1%)	0/6(0%)	0/6(0%)	0/6(0%)
12	SP4	CASYQTQTNSPRRARSV	0/22(0%)	2/22 (9.1%)	2/22 (9.1%)	0/22(0%)	1/22 (4.5%)	0/22(0%)	0/6(0%)	0/6(0%)	1/6(16.7%)
13	SP5	ARSVASQSIIAYTMSLGAENSVA	1/22 (4.5%)	5/22(22.7%)	1/22 (4.5%)	1/22 (4.5%)	1/22 (4.5%)	3/22(13.6%)	0/6(0%)	0/6(0%)	1/6(16.7%)
14	SP6	RALTGIAVEQDKNTQEVFAQV	2/22 (9.1%)	7/22(31.8%)	8/22(36.4%)	1/22 (4.5%)	4/22(18.2%)	1/22 (4.5%)	2/6(33.3%)	0/6(0%)	0/6(0%)
15	SP7	FAQVKQIYKTPPIKDFGGFNF	1/22 (4.5%)	4/22(18.2%)	4/22(18.2%)	1/22 (4.5%)	3/22(13.6%)	0/22(0%)	0/6(0%)	0/6(0%)	1/6(16.7%)
16	SP8	NFSQILPDPSKPSKRS	0/22(0%)	3/22(13.6%)	1/22 (4.5%)	0/22(0%)	4/22(18.2%)	0/22(0%)	0/6(0%)	0/6(0%)	1/6(16.7%)
17	SP9	KRSFIEDLLFNKVT	0/22(0%)	3/22(13.6%)	5/22(22.7%)	2/22 (9.1%)	3/22(13.6%)	2/22 (9.1%)	1/6(16.7%)	0/6(0%)	0/6(0%)
18	SP10	PELDSFKEELDKYFKNHTSPDVD	0/22(0%)	8/22(36.4%)	14/22(63.6%)	0/22(0%)	9/22(40.9%)	9/22(40.9%)	0/6(0%)	0/6(0%)	4/6(66.7%)
19	SP11	SPDVDLGDISGINASVV	0/22(0%)	8/22(36.4%)	11/22(50%)	1/22 (4.5%)	8/22(36.4%)	5/22(22.7%)	0/6(0%)	0/6(0%)	0/6(0%)
20	MP1	CDIKDLPKEITVATS	0/22(0%)	8/22(36.4%)	0/22(0%)	0/22(0%)	6/22 (27.3%)	0/22(0%)	0/6(0%)	0/6(0%)	0/6(0%)
21	MP2	QRVAGDSGFAAYSRY	0/22(0%)	10/22(45.5%)	0/22(0%)	0/22(0%)	4/22(18.2%)	0/22(0%)	0/6(0%)	0/6(0%)	0/6(0%)
22	NP1	ALLLLDRLNQLESKMSG	2/22 (9.1%)	4/22(18.2%)	1/22 (4.5%)	0/22(0%)	3/22(13.6%)	2/22 (9.1%)	0/6(0%)	1/6(16.7%)	0/6(0%)
23	NP2	NQLESKMSGKGQQQQGQTVTKKSA	1/22 (4.5%)	7/22(31.8%)	11/22(50%)	0/22(0%)	7/22(31.8%)	8/22(36.4%)	0/6(0%)	3/6(50%)	2/6(33.3%)
24	NP3	QTVTKKSAAEASKKPRQK	0/22(0%)	5/22(22.7%)	2/22 (9.1%)	0/22(0%)	3/22(13.6%)	1/22 (4.5%)	0/6(0%)	0/6(0%)	1/6(16.7%)
25	NP4	RGPEQTQGNFGDQELI	1/22 (4.5%)	0/22(0%)	4/22(18.2%)	0/22(0%)	1/22 (4.5%)	2/22 (9.1%)	0/6(0%)	0/6(0%)	1/6(16.7%)
26	NP5	AYKTFPPTEPKKDKKKK	1/22 (4.5%)	2/22 (9.1%)	2/22 (9.1%)	0/22(0%)	2/22 (9.1%)	0/22(0%)	0/6(0%)	0/6(0%)	1/6(16.7%)
27	NP6	KDKKKKADETQALPQR	0/22(0%)	2/22 (9.1%)	4/22(18.2%)	0/22(0%)	5/22(22.7%)	0/22(0%)	0/6(0%)	1/6(16.7%)	1/6(16.7%)
28	NP7	ADETQALPQRQKKQQTVTLLPAADL	1/22 (4.5%)	12/22(54.5%)	4/22(18.2%)	1/22 (4.5%)	6/22 (27.3%)	3/22(13.6%)	0/6(0%)	0/6(0%)	1/6(16.7%)
29	NP8	TLLPAADLDDFSKQLQQSMSSADS	0/22(0%)	6/22 (27.3%)	15/22(68.2%)	1/22 (4.5%)	13/22(59.1%)	5/22(22.7%)	0/6(0%)	1/6(16.7%)	3/6(50%)

Supplementary Table 4. Group and time point wise reactivity to 29 epitopes in plasma samples from COVID-19 patients

		Severe-COVID (Early)	Severe-COVID (Late)	Mild-COVID (Early)	Mild-COVID (Late)	Asymtomatic COVID (First)	Asymtomatic COVID (second)	Other HCoV controls	Healthy controls	SARS-CoV-1 controls
1	LGDELGTDPYEDFQ	0/22 (0%)	0/22 (0%)	0/22 (0%)	0/22 (0%)	0/6 (0%)	0/6 (0%)	0/10 (0%)	1/11 (10%)	0/11 (0%)
2	LYIDINGNLHPDSAT	0/22 (0%)	2/22 (10%)	2/22 (10%)	0/22 (0%)	0/6 (0%)	0/6 (0%)	0/10 (0%)	0/11 (0%)	0/11 (0%)
3	ATKYLVQQESPFVMM	0/22 (0%)	0/22 (0%)	0/22 (0%)	0/22 (0%)	0/6 (0%)	0/6 (0%)	0/10 (0%)	1/11 (10%)	0/11 (0%)
4	IVFDGKSKCEESSA	0/22 (0%)	0/22 (0%)	1/22 (5%)	1/22 (5%)	0/6 (0%)	0/6 (0%)	0/10 (0%)	0/11 (0%)	0/11 (0%)
5	LNDFSNSGSDVLYQP	0/22 (0%)	0/22 (0%)	0/22 (0%)	0/22 (0%)	0/6 (0%)	0/6 (0%)	1/10 (10%)	0/11 (0%)	0/11 (0%)
6	LSFCAFAVDAAKAY	1/22 (5%)	0/22 (0%)	0/22 (0%)	0/22 (0%)	0/6 (0%)	0/6 (0%)	0/10 (0%)	0/11 (0%)	0/11 (0%)
7	YDFGDFIQTTPGSG	0/22 (0%)	0/22 (0%)	0/22 (0%)	0/22 (0%)	0/6 (0%)	0/6 (0%)	1/10 (10%)	0/11 (0%)	0/11 (0%)
8	DFIQTTPGSGVPVV	0/22 (0%)	0/22 (0%)	0/22 (0%)	0/22 (0%)	0/6 (0%)	0/6 (0%)	1/10 (10%)	0/11 (0%)	0/11 (0%)
9	FLLKYNENGTITDAV	0/22 (0%)	1/22 (5%)	0/22 (0%)	0/22 (0%)	0/6 (0%)	0/6 (0%)	0/10 (0%)	0/11 (0%)	0/11 (0%)
10	GVLTESNKKFLPFQ	0/22 (0%)	0/22 (0%)	0/22 (0%)	0/22 (0%)	0/6 (0%)	0/6 (0%)	0/10 (0%)	0/11 (0%)	1/11 (10%)
11	YQTQTNSPRRARSVA	0/22 (0%)	1/22 (5%)	0/22 (0%)	0/22 (0%)	0/6 (0%)	0/6 (0%)	0/10 (0%)	0/11 (0%)	0/11 (0%)
12	NTQEVFAQVKQIYK	1/22 (5%)	0/22 (0%)	0/22 (0%)	0/22 (0%)	0/6 (0%)	0/6 (0%)	0/10 (0%)	0/11 (0%)	0/11 (0%)
13	**MADSNGTITVEELKKLLEQWN**	**1/22 (5%)**	**8/22 (37%)**	**0/22 (0%)**	**4/22 (19%)**	**0/6 (0%)**	**1/6 (17%)**	**0/10 (0%)**	**0/11 (0%)**	**0/11 (0%)**
14	KEPCSSGTYEGNSPFHP	0/22 (0%)	0/22 (0%)	0/22 (0%)	1/22 (5%)	0/6 (0%)	0/6 (0%)	0/10 (0%)	1/11 (10%)	0/11 (0%)
15	TLLPAADLDDFSKQ	1/22 (5%)	1/22 (5%)	0/22 (0%)	0/22 (0%)	0/6 (0%)	1/6 (17%)	0/10 (0%)	0/11 (0%)	0/11 (0%)
16	AADLDDFSKQLQQS	1/22 (5%)	0/22 (0%)	0/22 (0%)	0/22 (0%)	0/6 (0%)	0/6 (0%)	0/10 (0%)	0/11 (0%)	0/11 (0%)

Supplementary Table 5. Characteristics of selected sixteen IgM linear epitopes for detection of SARS-CoV-2 infection. Sequences (aa) are based on proteome of Severe acute respiratory syndrome coronavirus 2 isolate Wuhan-Hu-1 (Accession no. MN908947).

				Controls			SARS-CoV-2					
	Virus name	Epitope sequences	Protein name	Healthy	SARS-CoV-1	Other Human CoV	Asymptomatic (first)	Asymptomatic (Second)	Mild disease (first)	Mild disease (second)	Severe disease (first)	Severe disease (second)
1	Human coronavirus HKU1	MVNPSKGSSGLLITKLT	Nucleocapsid phosphoprotein	0/10 (0%)	0/11 (0%)	4/11 (37%)	0/6 (0%)	0/6 (0%)	0/22 (0%)	1/22 (5%)	0/22 (0%)	0/22 (0%)
2		VKQLPEQFDSLNLSAGTQ	Nucleocapsid phosphoprotein	1/10 (10%)	0/11 (0%)	2/11 (19%)	2/6 (34%)	0/6 (0%)	6/22 (28%)	6/22 (28%)	10/22 (46%)	11/22 (50%)
3		SLLATLDDPYVEDSVA	Nucleocapsid phosphoprotein	0/10 (0%)	1/11 (10%)	2/11 (19%)	0/6 (0%)	0/6 (0%)	4/22 (19%)	1/22 (5%)	5/22 (23%)	10/22 (46%)
4		MNKSFLPQFTSDQAV	Membrane glycoprotein	6/10 (60%)	6/11 (55%)	3/11 (28%)	6/6 (100%)	4/6 (67%)	19/22 (87%)	15/22 (69%)	18/22 (82%)	21/22 (96%)
5		WLNNFNVPSPLNWERKI	Spike glycoprotein	0/10 (0%)	0/11 (0%)	2/11 (19%)	0/6 (0%)	0/6 (0%)	0/22 (0%)	0/22 (0%)	1/22 (5%)	0/22 (0%)
6		ISASYRFVTFEPFNVS	Spike glycoprotein	2/10 (20%)	2/11 (19%)	4/11 (37%)	2/6 (34%)	0/6 (0%)	11/22 (50%)	10/22 (46%)	11/22 (50%)	10/22 (46%)
7		SRSFFEDLLFDKVKLS	Spike glycoprotein	1/10 (10%)	0/11 (0%)	1/11 (10%)	0/6 (0%)	1/6 (17%)	5/22 (23%)	3/22 (14%)	3/22 (14%)	4/22 (19%)
8		VDKQVILVSNNKEDFDIIQ	orf1ab polyprotein	1/10 (10%)	0/11 (0%)	1/11 (10%)	0/6 (0%)	0/6 (0%)	0/22 (0%)	2/22 (10%)	7/22 (32%)	11/22 (50%)
9		LLHSKVKVVPNLYVVV	orf1ab polyprotein	2/10 (20%)	1/11 (10%)	3/11 (28%)	0/6 (0%)	0/6 (0%)	1/22 (5%)	0/22 (0%)	2/22 (10%)	2/22 (10%)
10		FDLVQYDFTDYKLEL	orf1ab polyprotein	0/10 (0%)	1/11 (10%)	1/11 (10%)	0/6 (0%)	0/6 (0%)	4/22 (19%)	2/22 (10%)	7/22 (32%)	11/22 (50%)
11		PASQVIVNNYDKSAGY	orf1ab polyprotein	1/10 (10%)	0/11 (0%)	2/11 (19%)	0/6 (0%)	0/6 (0%)	0/22 (0%)	0/22 (0%)	4/22 (19%)	3/22 (14%)
12		CTGSPYIDDFNKIAS	orf1ab polyprotein	0/10 (0%)	0/11 (0%)	1/11 (10%)	0/6 (0%)	1/6 (17%)	6/22 (28%)	10/22 (46%)	11/22 (50%)	14/22 (64%)
13		CWETGKVKPPLNKNYVFTG	orf1ab polyprotein	2/10 (20%)	1/11 (10%)	1/11 (10%)	1/6 (17%)	2/6 (34%)	4/22 (19%)	5/22 (23%)	4/22 (19%)	5/22 (23%)
14		AKVRVDCYDKFKINDTTCKY	orf1ab polyprotein	0/10 (0%)	1/11 (10%)	2/11 (19%)	2/6 (34%)	2/6 (34%)	2/22 (10%)	1/22 (5%)	4/22 (19%)	4/22 (19%)
15		ISPYNSQNYVAKRVLGVQ	orf1ab polyprotein	1/10 (10%)	0/11 (0%)	1/11 (10%)	0/6 (0%)	0/6 (0%)	2/22 (10%)	2/22 (10%)	5/22 (23%)	6/22 (28%)
16	Human coronavirus NL63	EFRDYFNNNTDSIV	orf1ab polyprotein	2/10 (20%)	1/11 (10%)	2/11 (19%)	0/6 (0%)	0/6 (0%)	3/22 (14%)	3/22 (14%)	6/22 (28%)	8/22 (37%)
17		RKASGLFDAIWDAFVAAI	orf1ab polyprotein	2/10 (20%)	0/11 (0%)	1/11 (10%)	0/6 (0%)	0/6 (0%)	2/22 (10%)	1/22 (5%)	0/22 (0%)	1/22 (5%)
18		NVGEPIISQPSKLLNG	orf1ab polyprotein	0/10 (0%)	2/11 (19%)	1/11 (10%)	1/6 (17%)	1/6 (17%)	0/22 (0%)	0/22 (0%)	2/22 (10%)	1/22 (5%)
19		ISYLNRARGSSAARLEPC	orf1ab polyprotein	0/10 (0%)	1/11 (10%)	2/11 (19%)	0/6 (0%)	0/6 (0%)	0/22 (0%)	0/22 (0%)	0/22 (0%)	0/22 (0%)
20		CIKACEVVVTNLNKSAGWPLNKF GKA	orf1ab polyprotein	1/10 (10%)	1/11 (10%)	3/11 (28%)	0/6 (0%)	0/6 (0%)	1/22 (5%)	2/22 (10%)	1/22 (5%)	3/22 (14%)
21		VFINKYGLEDFNFEH	orf1ab polyprotein	0/10 (0%)	0/11 (0%)	1/11 (10%)	0/6 (0%)	0/6 (0%)	0/22 (0%)	0/22 (0%)	5/22 (23%)	5/22 (23%)
22		TVLNASIPESKPLADDDSA	Nucleocapsid phosphoprotein	6/10 (60%)	7/11 (64%)	3/11 (28%)	3/6 (50%)	3/6 (50%)	15/22 (69%)	17/22 (78%)	15/22 (69%)	21/22 (96%)
23		ASQEGVLSEREKLLHLI	Membrane glycoprotein	0/10 (0%)	0/11 (0%)	2/11 (19%)	0/6 (0%)	0/6 (0%)	0/22 (0%)	1/22 (5%)	1/22 (5%)	2/22 (10%)
24		QFGLQDGFYSANFLDDNVL	Spike glycoprotein	6/10 (60%)	3/11 (28%)	4/11 (37%)	1/6 (17%)	1/6 (17%)	6/22 (28%)	7/22 (32%)	8/22 (37%)	13/22 (60%)
25		CDCGSTKLPYYEFEKVHVQ	Spike glycoprotein	7/10 (70%)	0/11 (0%)	3/11 (28%)	1/6 (17%)	2/6 (34%)	8/22 (37%)	6/22 (28%)	12/22 (55%)	15/22 (69%)
26	Human coronavirus OC43	FDLVQYDFTDYKLEL	nsp9	0/10 (0%)	1/11 (10%)	1/11 (10%)	0/6 (0%)	0/6 (0%)	4/22 (19%)	2/22 (10%)	7/22 (32%)	12/22 (55%)
27		SQVIVNNYDKSAGYPF	nsp9	0/10 (0%)	0/11 (0%)	2/11 (19%)	0/6 (0%)	0/6 (0%)	0/22 (0%)	0/22 (0%)	6/22 (28%)	9/22 (41%)
28		LSTCDGQKFTDESFYKNMY	nsp9	1/10 (10%)	0/11 (0%)	2/11 (19%)	0/6 (0%)	0/6 (0%)	0/22 (0%)	1/22 (5%)	1/22 (5%)	6/22 (28%)
29		FPLKLAGTAVINLRAD	nsp13	0/10 (0%)	0/11 (0%)	2/11 (19%)	0/6 (0%)	0/6 (0%)	0/22 (0%)	0/22 (0%)	0/22 (0%)	0/22 (0%)
30		HATRDSIGTNFPLQL	nsp11	1/10 (10%)	0/11 (0%)	1/11 (10%)	0/6 (0%)	0/6 (0%)	5/22 (23%)	6/22 (28%)	2/22 (10%)	6/22 (28%)
31		CTGSPYIDDFNRIAS	nsp10	0/10 (0%)	0/11 (0%)	1/11 (10%)	0/6 (0%)	0/6 (0%)	2/22 (10%)	2/22 (10%)	3/22 (14%)	5/22 (23%)
32		AKVRVECYDKFKINDTTRKYV	nsp10	0/10 (0%)	2/11 (19%)	2/11 (19%)	1/6 (17%)	2/6 (34%)	1/22 (5%)	3/22 (14%)	2/22 (10%)	5/22 (23%)
33		ASSRSAIEDLLFDKVKLS	Spike glycoprotein	5/10 (50%)	5/11 (46%)	3/11 (28%)	1/6 (17%)	3/6 (50%)	13/22 (60%)	14/22 (64%)	13/22 (60%)	16/22 (73%)
34		SQVIVNNYDKSAGYPF	orf1ab polyprotein	0/10 (0%)	0/11 (0%)	2/11 (19%)	0/6 (0%)	0/6 (0%)	0/22 (0%)	0/22 (0%)	0/22 (0%)	0/22 (0%)
35		LSTCDGQKFTDESFYKNMY	orf1ab polyprotein	1/10 (10%)	0/11 (0%)	2/11 (19%)	0/6 (0%)	0/6 (0%)	0/22 (0%)	1/22 (5%)	1/22 (5%)	6/22 (28%)
36		AETQKATEEAFKQSYA	orf1ab polyprotein	0/10 (0%)	0/11 (0%)	1/11 (10%)	0/6 (0%)	0/6 (0%)	0/22 (0%)	0/22 (0%)	0/22 (0%)	0/22 (0%)
37		SWEIGKVKPPLNKNYVFTG	orf1ab polyprotein	0/10 (0%)	0/11 (0%)	1/11 (10%)	2/6 (34%)	2/6 (34%)	5/22 (23%)	4/22 (19%)	5/22 (23%)	6/22 (28%)
38		AKVRVECYDKFKINDTTRKYV	orf1ab polyprotein	0/10 (0%)	2/11 (19%)	2/11 (19%)	1/6 (17%)	2/6 (34%)	1/22 (5%)	3/22 (14%)	5/22 (23%)	8/22 (37%)
39		HATRDSIGTNFPLQL	orf1ab polyprotein	1/10 (10%)	0/11 (0%)	1/11 (10%)	0/6 (0%)	0/6 (0%)	6/22 (28%)	7/22 (32%)	2/22 (10%)	6/22 (28%)
40	Human coronavirus 229E	DVIDNEIIVKPNISLCVPL	orf1ab polyprotein	3/10 (30%)	3/11 (28%)	4/11 (37%)	1/6 (17%)	0/6 (0%)	9/22 (41%)	11/22 (50%)	16/22 (73%)	19/22 (87%)
41		KHSDFGLGDLVDSYFEND	orf1ab polyprotein	2/10 (20%)	0/11 (0%)	1/11 (10%)	0/6 (0%)	1/6 (17%)	10/22 (46%)	7/22 (32%)	15/22 (69%)	19/22 (87%)
42		CITSREVVVTNLNKSA	orf1ab polyprotein	0/10 (0%)	0/11 (0%)	3/11 (28%)	0/6 (0%)	0/6 (0%)	0/22 (0%)	0/22 (0%)	2/22 (10%)	1/22 (5%)
43		DVIDNEIIVKPNISLCVPL	orf1ab polyprotein	3/10 (30%)	3/11 (28%)	4/11 (37%)	1/6 (17%)	0/6 (0%)	10/22 (46%)	11/22 (50%)	16/22 (73%)	19/22 (87%)
44		KHSDFGLGDLVDSYFEND	orf1ab polyprotein	2/10 (20%)	0/11 (0%)	1/11 (10%)	0/6 (0%)	0/6 (0%)	12/22 (55%)	7/22 (32%)	15/22 (69%)	19/22 (87%)
45	SARS-CoV-1	LYEQVVMDYLDNLKPRVEAPKQ EEPPNTED	orf1ab polyprotein	4/10 (40%)	7/11 (64%)	3/11 (28%)	3/6 (50%)	4/6 (67%)	12/22 (55%)	14/22 (64%)	13/22 (60%)	14/22 (64%)
46		VSVSSPDAVTTYNGYLTSSSKTS EEHF	nsp3	2/10 (20%)	3/11 (28%)	0/11 (0%)	0/6 (0%)	0/6 (0%)	10/22 (46%)	10/22 (46%)	7/22 (32%)	12/22 (55%)
47		LGDELGTDPIEDYEQNWNTKHG SGALR	orf1ab polyprotein	3/10 (30%)	3/11 (28%)	2/11 (19%)	0/6 (0%)	0/6 (0%)	10/22 (46%)	13/22 (60%)	17/22 (78%)	18/22 (82%)
48		FTLRSITAQPVKIDNASPASTVH ATA	Orf3	0/10 (0%)	4/11 (37%)	0/11 (0%)	1/6 (17%)	1/6 (17%)	0/22 (0%)	1/22 (5%)	0/22 (0%)	1/22 (5%)
49		TIVVTEGDGISTPKLKEDYQIGGY SEDR	Orf3	0/10 (0%)	6/11 (55%)	0/11 (0%)	0/6 (0%)	0/6 (0%)	0/22 (0%)	0/22 (0%)	2/22 (10%)	1/22 (5%)
50		SSGVANPAMDPIYDEPTTTTSV PL	Orf3	1/10 (10%)	5/11 (46%)	0/11 (0%)	1/6 (17%)	0/6 (0%)	0/22 (0%)	2/22 (10%)	4/22 (19%)	8/22 (37%)
51		MADNGTITVEELKQLLEQWNLV IG	membrane glycoprotein	0/10 (0%)	7/11 (64%)	0/11 (0%)	0/6 (0%)	1/6 (17%)	1/22 (5%)	15/22 (69%)	4/22 (19%)	13/22 (60%)
52		TPKDHIGTRNPNNNAATVLQLP QGTTLPKGFYAEGSRGGS	Nucleocapsid phosphoprotein	0/10 (0%)	6/11 (55%)	1/11 (10%)	2/6 (34%)	3/6 (50%)	10/22 (46%)	16/22 (73%)	15/22 (69%)	22/22 (100%)
53		FSLDVSEKSGNFKHLREFVF	spike glycoprotein	1/10 (10%)	4/11 (37%)	0/11 (0%)	0/6 (0%)	0/6 (0%)	1/22 (5%)	2/22 (10%)	1/22 (5%)	4/22 (19%)

Supplementary Table 6. List of selected 53 IgG linear IgG epitopes for detection of HKU-1, NL63, OC43, 229E, SARS-CoV-1. Sequences (aa) are based on proteome of reference sequences from NCBI.
