# Supplementary material for: Immunoreactive peptide maps of SARS-CoV-2
Source: Commun Biol. 2021 Feb 12;4:225. doi: 10.1038/s42003-021-01743-9 (PMC7881038; doi:10.1038/s42003-021-01743-9)
Supplement: Supplementary file 2 — Description of Supplementary Files [file 42003_2021_1743_MOESM2_ESM.pdf]

Description of Additional Supplementary Files

File name: Supplementary Data 1

Description: Aggregate IgG peptide reactivity data

File name: Supplementary Data 2

Description: Aggregate IgM peptide reactivity data

File name: Supplementary Data 3

Description: MDS-IgG significant peptides- source data for Figure 1.A

File name: Supplementary Data 4

Description: Heatmap source data for Figure 1.B

File name: Supplementary Data 5

Description: List of all 163 IgG linear epitopes for detection of SARS-CoV-2 infection

File name: Supplementary Data 6

Description: Box plots IgG Source Data for Supplementary Figure 2

File name: Supplementary Data 7

Description: Reactivity with each of these 29 epitopes for the individual plasma samples

File name: Supplementary Data 8

Description: MDS IgM significant peptides source data for Supplementary Figure 3
