# Supplementary material for: Immunoreactive peptide maps of SARS-CoV-2
Source: Commun Biol. 2021 Feb 12;4:225. doi: 10.1038/s42003-021-01743-9 (PMC7881038; doi:10.1038/s42003-021-01743-9)
Supplement: Supplementary file 11 — Reporting Summary [file 42003_2021_1743_MOESM11_ESM.pdf]

nature research

Corresponding author(s): Nischay Mishra

Last updated by author(s): Dec 6, 2020

# Reporting Summary

Nature Research wishes to improve the reproducibility of the work that we publish. This form provides structure for consistency and transparency in reporting. For further information on Nature Research policies, see our Editorial Policies and the Editorial Policy Checklist.

## Statistics

For all statistical analyses, confirm that the following items are present in the figure legend, table legend, main text, or Methods section.

| n/a | Confirmed | |
|---|---|---|
| ☐ | ☒ | The exact sample size ($n$) for each experimental group/condition, given as a discrete number and unit of measurement |
| ☐ | ☒ | A statement on whether measurements were taken from distinct samples or whether the same sample was measured repeatedly |
| ☐ | ☒ | The statistical test(s) used AND whether they are one- or two-sided<br>*Only common tests should be described solely by name; describe more complex techniques in the Methods section.* |
| ☒ | ☐ | A description of all covariates tested |
| ☒ | ☐ | A description of any assumptions or corrections, such as tests of normality and adjustment for multiple comparisons |
| ☐ | ☒ | A full description of the statistical parameters including central tendency (e.g. means) or other basic estimates (e.g. regression coefficient) AND variation (e.g. standard deviation) or associated estimates of uncertainty (e.g. confidence intervals) |
| ☐ | ☒ | For null hypothesis testing, the test statistic (e.g. $F$, $t$, $r$) with confidence intervals, effect sizes, degrees of freedom and $P$ value noted<br>*Give P values as exact values whenever suitable.* |
| ☒ | ☐ | For Bayesian analysis, information on the choice of priors and Markov chain Monte Carlo settings |
| ☒ | ☐ | For hierarchical and complex designs, identification of the appropriate level for tests and full reporting of outcomes |
| ☒ | ☐ | Estimates of effect sizes (e.g. Cohen's $d$, Pearson's $r$), indicating how they were calculated |

*Our web collection on statistics for biologists contains articles on many of the points above.*

## Software and code

Policy information about availability of computer code

| | |
|---|---|
| Data collection | Peptide array images were scanned using Roche MS200 array scanner. |
| Data analysis | Rstudio version 1.2.5033, R version 3.6.2, GENEIOUS version 6.0, Custom code (Github: https://github.com/ciibioinformatics/COVID19_publication) |

For manuscripts utilizing custom algorithms or software that are central to the research but not yet described in published literature, software must be made available to editors and reviewers. We strongly encourage code deposition in a community repository (e.g. GitHub). See the Nature Research guidelines for submitting code & software for further information.

## Data

Policy information about availability of data

All manuscripts must include a data availability statement. This statement should provide the following information, where applicable:

- Accession codes, unique identifiers, or web links for publicly available datasets
- A list of figures that have associated raw data
- A description of any restrictions on data availability

The data shown in the manuscript is available as Supplementary Data.

# Field-specific reporting

# Life sciences study design

All studies must disclose on these points even when the disclosure is negative.

| | |
|---|---|
| Sample size | The sample size was determined/limited by available number of paired samples (early and late time point in COVID-19 group). Appropriate number of samples available to identify discrete immune-reactive epitopes for SARS-CoV-2 were analyzed using peptide arrays. Positive and negative controls for assay development and epitope identification showed a clear difference in reactivity pattern. |
| Data exclusions | All data was included in analysis. |
| Replication | Peptide arrays were designed to include whole proteomes of human coronaviruses from NCBI and VIPR databases. each array included variant of each peptide, even it is one amino acid mismatch. So with in each experiment every sample showed multiple replicates for IgG and IgM epitopes. |
| Randomization | The purpose of study was to identify reactive epitopes for SARS-CoV-2 so randomization was not performed. |
| Blinding | The purpose of study was to identify reactive epitopes for SARS-CoV-2 so blinding was not performed. |

# Reporting for specific materials, systems and methods

We require information from authors about some types of materials, experimental systems and methods used in many studies. Here, indicate whether each material, system or method listed is relevant to your study. If you are not sure if a list item applies to your research, read the appropriate section before selecting a response.

## Materials & experimental systems

| n/a | Involved in the study | |
|---|---|---|
| ☐ | ☒ | Antibodies |
| ☒ | ☐ | Eukaryotic cell lines |
| ☒ | ☐ | Palaeontology and archaeology |
| ☒ | ☐ | Animals and other organisms |
| ☐ | ☒ | Human research participants |
| ☒ | ☐ | Clinical data |
| ☒ | ☐ | Dual use research of concern |

## Methods

| n/a | Involved in the study | |
|---|---|---|
| ☒ | ☐ | ChIP-seq |
| ☒ | ☐ | Flow cytometry |
| ☒ | ☐ | MRI-based neuroimaging |

## Antibodies

| | |
|---|---|
| Antibodies used | Alexa Fluor® 647 AffiniPure Goat Anti-Human IgG, Fcγ fragment specific and Cy™3 AffiniPure Goat Anti-Human IgM, Fc5μ fragment specific are commercial secondary antibodoies. |
| Validation | Both antibodies have been used previously by different research scientific groups and mentioned in many peer reviewed publications. |

## Human research participants

Policy information about [studies involving human research participants](#)

| | |
|---|---|
| Population characteristics | Samples were de-identified before use. Hundred samples from COVID-19 patients and 32 were in control group (Non-COVID-19). |
| Recruitment | All samples were stored already. No enrollments were made for this study. |
| Ethics oversight | The study was approved by the Medical Ethical Committee of Sun Yat-Sen University (approval number 2020-060). |

Note that full information on the approval of the study protocol must also be provided in the manuscript.
